# Using focused ethnography in paediatric settings to explore professionals’ and parents’ attitudes towards expertise in managing chronic kidney disease stage 3–5

**DOI:** 10.1186/1472-6963-14-403

**Published:** 2014-09-18

**Authors:** Ruth Nightingale, Manish D Sinha, Veronica Swallow

**Affiliations:** NIHR Clinical Research Network: North Thames, c/o Somers Clinical Research Facility, Frontage Building, Level 1, Great Ormond Street Hospital for Children NHS Foundation Trust, Great Ormond Street, London, WC1N 3JH UK; Department of Pediatric Nephrology, Room 64, Sky Level, Evelina London Children’s Hospital, Guy’s and St Thomas’ NHS Foundation Trust, Westminster Bridge Road, London, SE1 7EH UK; Room 4.322a, School of Nursing, Midwifery and Social Work, Faculty of Medical and Human Sciences, Manchester Academic Health Sciences Centre, University of Manchester, Oxford Road, Manchester, M13 9PT UK

**Keywords:** Chronic illness, Child, CKD, Expert, Kidney, Long-term, Parent, Focused ethnography, Observation, Renal

## Abstract

**Background:**

Interactions between parents and healthcare professionals are essential when parents of children with chronic conditions are learning to share expertise about clinical care, but limited evidence exists on how they actually interact. This paper discusses the use of focused ethnography in paediatric settings as an effective means of exploring attitudes towards expertise.

**Methods:**

The paper draws on repeated observations, interviews and field-notes involving the parents of six children with chronic kidney disease, and 28 healthcare professionals at two, tertiary, children’s hospital-based units. Data were analysed using the Framework approach and the concepts of expertise and self-management.

**Results:**

Our study highlighted rewards and challenges associated with focused ethnography in this context. Rewards included the ability to gain a richer understanding of the complex phenomena of mutual acknowledgement of expertise that occurs during parent/ healthcare professional interactions. Challenges related to gaining informed consent and ensuring potential participants had an adequate understanding of the purpose of the study. Two dimensions of parental expertise around their child (personal and clinical) were evident in our data. Parents’ and professionals’ expertise about the child and their condition was acknowledged and exchanged as parents learnt to share clinical-care with the multi-disciplinary team. Healthcare professionals acknowledged parents’ need to understand aspects of each of the eight disciplinary knowledge bases relating to their child’s management and recognised parents’ expert knowledge of their child, found ways to mobilise this knowledge, and wove parents’ expertise into the management plan. Parents spoke of the degree to which their own expert knowledge of their child complemented healthcare professionals’ clinical knowledge. However, ambivalence around expertise was evident as both parents and healthcare professionals questioned what the expertise was, and who the expert was. Our discussion focuses on the ways healthcare professionals and parents share expertise around the child’s condition as parents take on responsibility for home-based clinical care.

**Conclusions:**

Our findings point to focused ethnography being an effective way of capturing new insights into parent and professional interactions in a paediatric setting and mutual acknowledgement of expertise; these insights may help redress the reported limitations of previous, retrospective studies.

## Background

Childhood chronic kidney disease (CKD) is a complex set of conditions that involves healthcare professionals (HCPs) and parents sharing management [1–4]. Children and young people (children) with CKD stages 3–5 require particularly complex medical and dietary regimens, and their disease may progress over time from stage 3 to stage 5. One risk factor for disease progression is poor adherence to treatment regimens which may be linked to parents having limited access to or poor understanding of care-giving information. However, when the child’s condition is stable parents may deliver the majority of clinical care at home [3].

Little evidence exists to inform our understanding of the interactions between parents and HCPs as parents learn to master the clinical skills to manage their child’s CKD at home. This gap in the evidence may exist because of the perceived difficulty and ethical concerns around obtaining consent and safeguarding privacy when conducting research that involves observing interactions in clinical settings [5, 6]. Ethnographic research is one methodological approach that can address these challenges but it is argued that it has been rarely used as a methodology for the in-depth study of paediatric healthcare issues in the context in which they occur, due to these reported concerns [7]. Whilst ethnography remains challenging in paediatric settings and in the wider healthcare sector, there is evidence of use of this approach in a wide range of other settings and with a range of participants [8, 9]. However, with careful consideration, discussion with stakeholders, and appropriate approvals, users of ethnographic research findings in paediatric settings can be reassured about the rigour of studies using this methodology.

In this paper we discuss phase 3 of a three phased study in which we sought to obtain a detailed insight into the way multidisciplinary teams (MDTs) in a network of 12 UK children’s kidney units teach parents to share CKD management in preparation for home-based clinical care [10]. We previously outlined the way parents and professionals in this study negotiated common ground in shared-care, as part of this process we noted that professionals drew on parents’ expert knowledge of their child [11]. This paper explores and discusses how we used a focused ethnographic methodology as an effective means of exploring professionals’ and parents’ attitudes towards expertise and of considering the strengths and limitations of using observational methods in an acute child-health care setting.

Although reports of ethnographic and observational research in child-health are beginning to emerge, we believe this is the first time that a longitudinal, prospective, focused ethnographic approach designed to explore ‘live’ communications as parents learned to share management has been used; the study has started defining this aspect of communication which is one of the vital ingredients of the complex intervention [12] of parent teaching and learning in CKD care.

### The rationale for using focused ethnography

Parents are increasingly responsible for managing complex aspects of clinical care at home for children with long-term conditions, yet few researchers have attempted to explore the actual process of teaching and learning within HCP-parent encounters in acute child-health settings. A limitation of previous studies is that they were either retrospective or used participants’ reports of their experience rather than observation, and so were unable to focus on actual encounters between HCPs and parents at times when parents were learning to become home-based clinical caregivers. Therefore, detailed, prospective research that investigates the ways HCPs promote learning from early in the parents’ clinical care-giving journey is needed. This has the potential to inform MDTs about the factors that are seen to be important in HCP-parent interactions as HCPs teach parents to deliver safe and effective home-based clinical management, thereby contributing to optimum clinical outcomes for children.

We, therefore, used a prospective methodological approach that would enable us to contribute to the evidence base on the way HCPs teach parents of children with CKD, and the way parents learn to become home-based clinical care-givers. We sought to shift the focus away from that reported in the literature that investigated parents’: management of conditions such as CKD, [13, 14], work associated with managing chronic illness [15], information needs [16–18]; and roles [19–22]; notably these studies looked broadly and usually retrospectively at parents’ management activity.

A focused ethnographic approach [23] was utilised in this phase. Ethnography aims to understand the cultural meanings people in a specific culture use to interpret their experiences [24, 25] whereas focused ethnography has emerged as a method for applying ethnography to ‘focus on a distinct issue or shared experience in cultures or sub-cultures in specific settings…..rather than throughout entire communities’ [23:36]. A central assumption of this approach is that to understand what people are doing and why: ‘…one needs to understand the meanings involved: how they interpret and evaluate the situations they face, and their own identities’ [25:168]. Focused ethnographic research often uses small sample sizes as its aim is to explore participants’ beliefs and practices, viewing them within the context in which they actually occur rather than aiming to produce findings which can be generalised [26].

Using this approach enabled us to gain a detailed insight into the social context in which MDTs support the learning process of parents, and to report on the actual parent-HCP experience of sharing knowledge and skills around clinical management. These insights are generally not as accessible through other research approaches such as those widely reported in the literature (e.g. quantitative methods or qualitative accounts that are retrospective).

Focused ethnographic research involves generating data by observing a group or everyday social setting [26, 27]. Observing events in their natural social context can provide valuable insights, in particular into the relationships and communications between parents and HCPs and the cultural meanings they use to understand these social processes. Though this type of research is recognised as particularly valuable in researching child healthcare as it aims to create more balanced relationships between researchers and participants compared to other methods [24], its use has been limited, especially in the area of shared management of CKD and in acute paediatric settings.

Ethnographic fieldwork was undertaken in one hospital renal unit to explore the experience of long-term renal illness from the perspective of children and young people [1]. Through using ethnographic methodologies, new information was uncovered around children’s compliance with treatment and adolescents’ experiences of renal replacement therapies. Other studies which have used observational methods to focus on parents’ experiences in acute settings have explored the level at which parents participate in decision-making whilst their child is in hospital, and relationships between HCPs and parents in neonatal intensive care units [28, 29]. Coyne [30] explored children’s, parents’ and nurses’ views on participation in healthcare on paediatric wards using observational methods and interviews. She found consultation with children about their care was widely believed to be important, but in reality children’s views were underused; however, only the interview data appeared to be included in the analysis. Observational methods were utilised in children’s outpatient settings to look at exchange of information between children, young people and staff [31] and how HCPs share responsibility with children and parents [32].

As illustrated by these studies, ethnographic and observational research can provide valuable insights, in particular into the relationships and communications between children, parents and HCPs. However, only one of these studies specifically focused on children with CKD and none focused on the actual interactions between HCPs and parents as they begin to share clinical management.

Through utilising focused ethnography, we found we were able to explore HCPs’ and parents’ attitudes towards expertise; therefore we adopted an analytical framework based on concepts of expertise and self-management [33]. These concepts are of particular relevance in healthcare for children and adults living with chronic conditions, where the White Paper, Our Healthier Nation – Saving Lives [34] outlines a philosophy which recognises how a chronically ill individual’s expertise and knowledge about their own condition, should be utilised [35]. This concept of an expert patient as someone who is empowered to self-manage their own condition, and on an equal footing with HCPs [36], is also relevant when considering parents of children with chronic conditions such as CKD as they are often required to deliver complex clinical care to their child at home.

Expertise in healthcare has various definitions. Kirk [19] suggests that parents of children with chronic illness view their own expertise as consisting of two different types of knowledge; the specialised medical knowledge associated with their child’s condition which they have acquired from HCPs, and the experiential knowledge obtained through caring for their child on a daily basis. The differences between parental and HCP expertise can result in difficulties integrating these two different knowledge bases and lead to HCPs failing to recognise and utilise parents expertise [37–39]. Both Thorne [40] and Kirk [19] argue that the scientific expertise of HCPs is privileged during interactions between parents and HCPs, as medical expertise is viewed as more reliable and valid than that of the lay person [35]. A recent literature review by Smith et al. [41] confirmed the overwhelming picture presented in research evidence that parents perceive their expertise is not always valued and HCPs’ attitudes and willingness to collaborate in relation to care-decisions is variable and inconsistent. Smith et al. [41] concluded that further research is necessary both around the concept of the expert parent and how parents develop the expertise to manage their child’s condition. They believe this would ensure parents get suitable support when learning to share clinical care. The contemporaneous study reported here addresses this gap by exploring parents and HCPs attitudes towards expertise as parents learned the skills and knowledge needed to carry out clinical care at home for their child with CKD stage 3–5.

## Methods

### Study overview

This paper focuses on the third phase of a mixed-methods study in 12 of the 13 British children’s kidney units (10 in England, 1 each in Wales, Scotland and Northern Ireland). As previously mentioned, the wider study sought to obtain a detailed insight into the way MDTs in the 12 units teach parents to share CKD management in preparation for home-based clinical care. This aspect of the study is described in detail elsewhere [42] but in summary, in phase 1 we used a questionnaire to survey HCPs’ teaching interventions in the 12 units, followed by a second phase of qualitative interviews with 112 HCPs from eight different disciplines to retrospectively explore HCPs’ accounts of their parent-teaching strategies. Three key themes emerged: (i) initiating parents’ knowledge and skill development, (ii) monitoring parents’ knowledge and skill development, and (iii) MDT working. In phase 3, we then identified two units for focused ethnographic exploration [23, 43] of HCP-parent interactions as parents learned to share clinical management. The criteria for selection of the two units was that they needed to have patient caseloads that were likely to yield a sample that allowed us to achieve maximum sampling variation.

### Study setting

The study setting involved two tertiary, children’s hospital-based units each comprising a ward and an outpatient department. Children attending the unit for in- or out-patient care and their parents/carers are supported by a range of HCPs with expertise in managing CKD stages 3–5. Some professionals may see families in both the ward and outpatient setting as well as visiting the family home to teach or reinforce clinical skills and/or to offer social and psychological support as needed. The researcher (RN, an Occupational Therapist who had worked with children and young people and had practiced within the NHS for over 10 years, but who had no experience of renal units) initially spent time in the two units to become familiar with the settings. This enabled her to meet the staff involved with study families and to explain the aims of the study, thereby enabling her to familiarise herself with the culture and layout of the units.

### Study procedure

Six case-studies were undertaken, three studies in each of the two units. Potential index case patients were identified by the Principal Investigator (PI) in each unit. Parents of the first six patients meeting the purposive sampling criteria based on the child’s age, sex, ethnicity and clinical interventions parents needed to learn (e.g. these included administering complex medications, dietary supplements, feeding using gastrostomy or naso-gastric tube, delivering home dialysis, monitoring diet and fluids, recognising subtle clinical changes, recording clinical observations, acting on results, accurately communicating observations/actions to HCPs) provided written consent. The index-cases were two boys (aged 5 months and 8 years) and four girls (aged 3, 11, 12 and 15 years); five were White/British, one was South Asian. In total, 18 family members (patients, mothers, fathers and grandparents) participated; each case-study lasted six months with no attrition during the study period.

A combination of snowball and convenience sampling [44] was used to identify the HCPs involved in the management of each case; at the start of each case-study access negotiations commenced on an individual basis with identified HCPs. A total of 28 HCPs participated (representing the disciplines of dietetics, nursing, medicine, pharmacy, play-therapy, social work and therapy). As parents learned selected skills and knowledge, interactions between HCPs and parents were explored using a combination of data collection methods including:

1. **86 observations of interactions between parents and HCPs.** Observations took place over a six month period for each family participating in the study and occurred either in the outpatient clinic, renal ward or at families’ homes. Between 6 and twenty observations were carried out in relation to each family and each observation lasted between 20 minutes and 3 hours. An initial observation occurred at the start of data collection after which subsequent interactions were sampled to observe, with the aim of collecting pertinent data but also related to what was achievable by one researcher within the time frame of the study.
2. **41 semi-structured, individual qualitative interviews with mothers, fathers and HCPs following selected observations.** Interviews explored parents’ and HCPs views of the effectiveness of observed communications and allowed more detailed exploration of how HCPs supported parents to share management of their child’s condition. HCPs were asked to focus on the participating family, though where relevant to the focus of the study, they discussed their wider experiences of supporting parents to undertake home-based clinical care.
3. **Selected case note and documents reviews.** Children’s case notes and relevant documents (e.g. competency checklists used when parents were learning home dialysis) were reviewed as part of data collection. This involved examining, and recording information on parents’ teaching and learning needs, progress and achievement, comparing this with observation and interview data and verifying details of conditions, medications or investigations mentioned during observations and interviews. Data from casenote and document reviews were analysed and the results triangulated with observation and interview data to inform subsequent data collection.

Detailed information was gathered on how individual HCPs supported parents, and how parents learned to develop the skills and knowledge needed to carry out home-based, clinical care-giving tasks. As data collection proceeded, the inquiry became progressively focused on specific research questions. This allowed for strategic data collection which meant answers to questions could be pursued more effectively by the researcher, and tested against existing data and research literature. Discussion with the study steering group about preliminary data also resulted in increasingly focused data collection.

Data management and analysis involved Framework Technique and use of MICROSOFT Excel [45–47]; this is an iterative-inductive approach whereby researchers move back and forwards between idea, design data collection, theory and findings. During preliminary analysis an initial coding framework comprising of descriptive and analytic codes was developed and collaboratively agreed. This framework was applied to the interview transcripts and field notes and refined as analysis proceeded. Data were then sorted and synthesised. The essence of participants’ key terms and phrases were retained as much as possible until the later interpretative stage of the analysis when four different categories began to emerge: (i) HCPs enabling and promoting parents’ clinical role, (ii) parents’ cautious acceptance of the clinical role, (iii) blended expertise around the child; and (iv) ambivalence in teaching and learning encounters. During this process it became clear that HCPs actively and consistently promoted parents’ clinical roles and expertise at the same time as they assessed and managed the child’s clinical condition.

### Reflections on the experience of using focused ethnography in a paediatric setting

Ethical approval was obtained prior to conducting the study and all participants provided informed written consent. When seeking Research Ethics Committee (REC) and NHS Trust Research and Development Department approvals for this study, we were encouraged that no concerns were expressed by the committees about the proposed study; in fact the REC commended the applicants for their detailed consideration of ethical issues. However, given that focused ethnography is under-utilised in acute child health settings, and in order to contribute new knowledge to this emerging form of research practice in this context, discussion in the remainder of this section will focus on the challenges and rewards of using this methodology, in particular when negotiating access and establishing this type of study in the clinical setting.

### Challenges

#### Negotiating access

Potential study families were identified by the PIs (a nurse and a doctor who worked in the two respective units); as knowledgeable insiders, they acted as gatekeepers and advised and supported the recruitment process. As the two units had participated in Phases 1 and 2 of the study, and the researcher (RN) had spent time meeting with MDT members before Phase 3 data collection started, many HCPs were already aware of the study. Though RN outlined the aims of the study to families and HCPs as part of negotiating consent, focused ethnographic research requires ‘improvisational strategies that are sensitive to multiple and shifting sources and types of available data, as the researcher’s understanding of relevant phenomena and relationships unfold over time’ [6:22]. For example, our understanding of the concepts of ‘teaching’ and ‘learning’ shifted over the course of the project as it became apparent that all participating MDT members were involved in these processes, but in different ways and using varied terminology to describe their contributions. Similarly, parents used different terminology to describe the support they received from the MDT when learning to deliver home-based clinical care, which meant RN often needed to use less formal terms than ‘teaching’ and ‘learning’ when asking them to describe their experiences during interviews.

Yang and Fox [48] suggest that ethnographic research can sometimes be confusing to those who are unfamiliar with its methods and aims and the role of the researcher can be unclear to participants [49]. In our study, RN initially found that some HCPs were interested in hearing how parents evaluated the service provided either by themselves or the MDT which tested RN’s need to maintain confidentiality; for example, some HCPs questioned what notes RN was taking during observations. In these situations, RN explained that she was noting down the details of what was happening in the interaction (e.g. what was being said, who was doing what) but did not share the actual notes she had made. Additionally, RN sometimes used these situations as an opportunity to informally discuss an aspect of the observed interaction with the HCP; for example, how the HCP had used humour as part of their communication with parents, which helped with gaining further insight.

Focused ethnographic research can also raise challenges in terms of gaining informed consent and ensuring potential participants have an adequate understanding of the purpose of the study [49]. Clarifying the aims of the research, the role of the researcher and reconfirming consent all needed to be repeated frequently throughout the study, and took time and the development of trustful and confidential relationships between RN and participants. Carrying out observations within a busy hospital setting meant that gaining consent could be difficult at times. For example, HCPs who RN had not previously met and therefore had not had the opportunity to explain the research to, would join outpatient appointments where RN was present as an observer, which meant explaining the research and obtaining written consent would sometimes occur retrospectively. On these occasions, RN reassured the HCPs concerned that any data collected which related to them, would not be used if they chose not to consent; no HCPs refused consent but a few who had given verbal consent did not actually provide written consent, despite RNs attempts to obtain this, due to their clinical workload taking a priority. In those instances, if RN was unable to obtain their written consent within the study timescale, any data collected regarding their interactions were not used.

#### Establishing a focused ethnographic study

Once initial access to the two units had been negotiated, the process of establishing a focused ethnographic study in a paediatric setting provided both opportunities and challenges. One concern raised in relation to this type of research is that the presence of a researcher may potentially disturb natural interactions and trigger a change in the behaviour of those being observed [50]. Carnevale et al. [6] expressed concerns that observations could be obtrusive and/or psychologically distressing for some participants. During our research observations, a red card was available for participants to signal if they felt uncomfortable and wanted RN to leave an observed situation. In addition, support from a Clinical Psychologist on the research team was available to those who experienced any distress from participating in the study. Neither of these measures were utilised by any participants, though RN decided to leave one observed situation when the child became distressed during a clinical care-giving task as it did not feel comfortable or appropriate for RN to continue observing. Additionally, one of the families agreed to RN observing interactions in relation to one aspect of the child’s renal care, but the mother asked her not to observe other appointments as both parents felt the child may become upset by the discussion taking place and they wanted to make sure that they would be able to give their full attention to ensuring their child’s well-being.

The aims of ethnographic research will influence the role adopted by the researcher in the setting, ranging from observer through to full participant [27], though in focused ethnographic research, the researcher tends to occupy a field-observer role as opposed to the participant role in traditional ethnographic research [23]. In our study, RN aimed to be minimally intrusive when observing interactions between HCPs and parents; for example, by choosing to sit slightly outside of the interaction and only participating minimally (i.e. laughing in response to jokes, making eye contact, smiling during conversations, taking discrete notes). O’Reilly [24] explored the dilemmas posed by ethnographic research, suggesting that the researcher trying to act as if they were not present during an observation would have ‘effects’ on the interactions, however, she does not describe what these ‘effects’ are, or for example, whether they are negative or positive. On many occasions in our study, this role of minimally intrusive observer could be achieved, especially as families and HCPs were reported to be used to observers being present (e.g. trainee and visiting HCPs), with some HCPs commenting that in time they ‘forgot’ that RN was present.

This ‘forgetting’ that they were being studied may have been beneficial to us as it meant typical behaviour could be observed, but this also potentially raises ethical dilemmas if it can result in participants disclosing information to the researcher that they may have intended to share only with the HCP involved in their child’s clinical care [6]. On a few occasions, it seemed parents may have forgotten that RN was a researcher and would either ask her health-related questions about their child’s care, or share concerns with her when a HCP was not present. In each situation RN’s observer role was challenged and in thinking about what was in the child’s and families best interests the role became more participatory, as RN either questioned and/or encouraged the parent to ask the question or share the concern with the HCP working with them. In these situations, the parent did always repeat the information to the HCP; however, if they had chosen not to, RN’s role would have been further challenged, as to act in the child’s and/or families best interests RN would have needed to have actively intervened and informed the MDT of the concerns. Additionally, as Lambert et al. [50] suggest, the dividing line between informal discussions with participants and more formal interviews is hard to differentiate at times, reinforcing the need in our study for RN to regularly remind participants of her role as a researcher.

There were other occasions when RN’s role became more participatory in response to specific situations and/or practicalities. For example, in some clinic rooms, the limited space and seating meant RN had to physically occupy a more participatory role in the interaction. With one set of parents, whose first language was not English, one of the HCPs was able to speak with them in their first language and then translated into English for RN’s benefit. The accuracy of the translation and the effect this had on the interaction with the parents is uncertain and could be viewed as a potential limitation. At other times during interactions, it was unclear whether RNs presence had an effect on the interaction; for example, some HCPs gave positive feedback to families by commenting to RN on how they thought the parents were learning, as illustrated by the following field data excerpt:

*Parents are learning with a nurse how to complete home dialysis. The mother is practicing on a teddy bear, connecting it to the dialysis machine. The father is watching what the mother is doing. The nurse comments to me [RN] how the session is very informal and relaxed, how the parents are chatting whilst doing tasks. The nurse thinks the parents have learnt quickly and have confidence as they are able to talk whilst doing tasks. She explains how other parents are not usually able to chat and have to keep quiet to concentrate. The father jokes about not being ‘normal’. Nurse says to the parents: ‘It’s a compliment’.*

All of these situations presented some challenges to RN’s position within the interaction, and the benefits of being a HCP with experience in child health helped to guide RN’s decision-making [6]. However, being a HCP initially presented personal challenges for RN in the transition to researcher as she was used to actively working as part of a MDT around a child’s healthcare (although not with children being treated for CKD and not in the study site), rather than being solely an observer of interactions between HCPs and parents. This may have explained why at times RN edged towards ‘going native’ which occurs when the observer starts to forget their role as an outsider and starts to take on a more participatory role [27]; for example, participating in interactions between HCPs and parents and temporarily losing the detached stance required to critically analyse the interactions. The importance of making detailed, anonymised field notes after observations, and of discussing these with other members of the research team helped ensure that RN was able to re-occupy the role of observer.

### Rewards

Some of the challenges presented by using a focused ethnographic methodology and the approaches used to overcome these challenges have been explored. The rewards of utilising focused ethnographic research methods will now be examined, and in particular how this was effective in capturing focused insights into parent/ HCP interactions and mutual acknowledgment of expertise.

Carnevale et al. [6] suggest that observation techniques are more suitable for examining the complex phenomena regarding children’s healthcare and their families than more structured research methods. This is particularly relevant when exploring the experiences and attitudes towards expertise of parents and MDT members as parents learn to deliver home-based care, as it is unlikely such rich data could have been gathered through interviews only. Focused ethnographic methods can result in researchers gaining access to settings and situations which often are not open to researchers; this allows data to be elicited and insights fostered which are not as accessible via other research methods [6, 24]. The richer understanding gained by drawing on multiple data sources such as those in our study (i.e. observations, interviews, case note and document reviews) can result in a unique emphasis when representing participants’ perspectives and the ‘social forces which guide and give meaning’ to the participants’ actions and experiences [48]. We assert that through using a focused ethnographic approach we were able to elicit insights into the interactions between parents and HCPs when parents were learning to deliver home-based clinical care, that are not readily available when using other methods.

Focused ethnographic research methods aim to create symmetrical relationships between researchers and participants and can be more ‘status levelling’ than other methods because they are flexible, allow time for relationships to develop between the research participants (e.g. parents and HCPs) and the intervening researcher, and incorporate a relational context [6, 24, 25, 48]. In focused ethnographic studies the researcher is required to spend time in the setting, and through this sustained presence, the ‘reactivity’ of participants to the researcher is believed to be reduced [6]. The prolonged time spent by RN in the two units, in combination with the busy real-life environment, is likely to have meant participants would have found it difficult not to ‘resort’ to their typical behaviour in RN’s presence. Hallstrom et al. [51] suggest observation can expose the differences between idealised and reality-based statements; such as the discrepancies between what participants report in interviews and what the researcher observes, and in addition can discover behaviour and practices which participants themselves are unaware of. The benefits of conducting interviews after selected observations meant RN could explore with participants their understanding of the observed interactions and, where relevant, explore the differences between observations and reports.

Through utilising focused ethnography, we suggest that we were able to gain a richer understanding of the complex phenomena of mutual acknowledgement of expertise that occurs during parent/HCP interactions. Our data suggest that HCPs acknowledged parents’ need to understand aspects of each of the eight disciplinary knowledge bases (e.g. clinical psychology, dietetics, medicine, nursing, pharmacy, play work, social work, therapy) relating to their child’s management; however, HCPs also recognised parents’ expert knowledge of their child, found ways to mobilise this knowledge, and wove parents’ expertise into the child’s clinical management plan. Parents also spoke of the degree to which their own expert knowledge of their child complemented HCPs’ clinical knowledge. This blended expertise comprised of a clinical domain where professionals and parents try to use their respective expertise to manage the child’s clinical needs. However, uncertainties do exist around the interactions between parents and professionals. Data which illustrates how expertise is acknowledged and shared, will now be presented and discussed.

## Results

### Acknowledging and sharing expertise

As parents and HCPs became familiar with each other’s knowledge of issues around the child, new relationships developed that helped them recognise mutual expertise. Individuals’ accounts in interviews often alluded to a mutual recognition of their respective forms of expert knowledge of the child and the child’s condition. This was supported by data collected during observations as illustrated by the following field note data which indicate the ways in which HCPs acknowledged parents’ expertise and previous experience of a long-term condition to help illustrate a point relating to the child’s current management:

*Later on in the appointment, the child’s father is making comparisons between using the dialysis machine with their previous experiences with their child’s feed pump. The nurse demonstrates how to check the line and seals on the dialysis fluid bags and advises: ‘It’s like you do with your feed pump’*

1. *The dietician is talking with the mother about how the child’s feed has been changed. The dietician says: ‘You know how to do the feeds. You’ve done it many, many times before. It’s a shame there are so many ingredients’. The mother replies: ‘I’m used to it’*
2. *Nurse is showing parents how to deal with alarms on the dialysis machine. Mother says: ‘We’ve had that with the feed pump (alarms going off). We sort it out, go back to bed and then 3 minutes later we have to get up again. We better make sure we have our glasses this time. We both wear contact lenses. We could be pressing any buttons!’.*

The fieldnote data also indicate how parents drew on their previous experience and expertise in managing their child’s condition to inform their own learning; for example, recognising the importance of having their glasses close by during the night in case they need to get up to tend to their child. The parents’ ability to joke about this issue suggests that the acknowledgement of their expertise contributes to a feeling of confidence in their ability to manage home-based clinical care.

Part of the expertise of HCPs was the ability to enable parents to reveal and work with their own expertise around their child. Examples of this are when a HCP asked a parent for advice, and where a parent appeared to take on the HCP’s role of becoming ‘teacher’ and/or ‘supervisor’ for other family members. This acknowledgement by HCPs’ of parents’ expertise was evident in many observations as illustrated by this excerpt from a field note:

*Home visit: Both of the child’s parents have been learning how to do home dialysis with their child; however, the mother is the main carer as the father works long hours. Conversation between mother and nurse about how the mother can support the child’s father to maintain his skills and competence in dialysing their child when he’s not doing it each day. The nurse says to the mother: ‘It might be worth you showing dad how you do [a procedure to check the dialysis machine]. You’re more thorough than me. If you have found a way to do it, then show him that way. The circuit is still new to me, so any tips are useful’.*

Field note data from a clinic appointment (during which the child was being dialysed) supports the idea that parents were viewed as the expert in some aspects of their child’s clinical care:

*Nurse checks the child’s blood pressure and then says to the mother: ‘Her blood pressure is 58, how do you manage that at home?’*

*Mother: ‘I either turn it down (the machine) or take her off’ (child is disconnected from the dialysis machine).*

*Mother and nurse agree to take the child off dialysis machine. Doctor says to the mother: ‘You could teach the girls! [other nurses in the team]. We need a dialysis nurse!’*

*Mother laughing and says: ‘It’s too long a commute!’*

Parents had their own knowledge of the child that could be supplemented over time by the expertise that they developed around condition management. Both parents and HCPs discussed parents’ experiences of sometimes knowing more than HCPs about details of the child’s clinical care. This was observed in different settings, for example when a child was admitted to a renal ward and the parent gave advice to ward staff (i.e. usual timings of their child’s medications).

The data suggest that HCP and parent forms of expertise are shared and blended to manage the child’s clinical needs. Through providing explanations to parents, HCPs aimed to continuously involve parents and promote their understanding of the consequences for the child. In addition, HCPs would prepare parents to share the child’s on-going clinical management where appropriate. As already mentioned, this shared care was likely to involve parents taking on responsibility for a wide range of home-based clinical care; the skills and knowledge that parents needed to take on this responsibility could reflect the distributed expertise of the entire MDT. The data indicate that HCPs were often concerned about the best way to explain these issues to parents. On the one hand, HCPs recognised the child-specific expertise that parents brought to the clinical role and wanted to ensure that parents could use this knowledge as they developed the expertise to safely manage their child’s condition at home; on the other hand they wanted parents to know when and how to seek assistance with their clinical role.

### Ambivalence around expertise

Though both HCP and parent expertise was mutually acknowledged within interactions, ambivalence around the concept of expertise was also evident within teaching and learning encounters. Through utilising focused ethnographic methods, it was possible to uncover the uncertainties experienced by MDT members and parents when engaging in teaching and learning. For example, there was evidence of ambivalence around *what* the expertise was and *who* was the expert as illustrated by this field note data where a parent questioned the technique she needed to learn to measure her child’s blood pressure:

*Mother: ‘I spoke with my mum’s nurse and asked why we couldn’t use one of those machines to record it electronically’.*

*Nurse: ‘We use these [she gestures towards the machine on the desk which takes blood pressure manually], not the electronic, as it’s proven in research, that if you have abnormal blood pressure, then it tends to be a higher reading on the electronic, it’s more accurate to use this one (the manual machine). We need a really accurate reading when you do dialysis. [Nurse turns to child to include her in the conversation]. At the moment your kidneys are working and you are still producing urine, but they may stop and so the [dialysis] machine would have to take off the fluid. We would need a very precise blood pressure reading to make sure enough fluid is coming off’.*

*Mother: ‘My mum’s nurse was saying many nurses can’t do it this way!’ [measuring blood pressure manually rather than electronically]*

There was recognition amongst MDT members and parents that some HCPs used different techniques to other HCPs when carrying out the same clinical task. For some parents this caused uncertainty, as they were unclear about which technique they should be using, as one mother explained during an interview:

*“It’s not always the same nurse, all nurses do things differently and I’ve learnt that, but the way we’re taught, or the way we’ve been shown, I’d like to be shown it again. If there’s anything shown different and then having to question it, and then because, I get a little bit, well, I don’t really want to question it, because they know what they’re doing” (Mother_6)*

MDT members gave examples of how HCPs might create additional difficulties for parents taking on clinical care by using different approaches to support parents’ learning; for example, providing information about a treatment (e.g. medication) in alternative ways, and assessing whether a family is coping with delivering care-giving from their own particular perspective on coping. In some cases it appeared this could create ambivalence around the concept of expertise. Some HCPs recognised these ambivalences and reflected on whether it would help parents’ learning if HCPs’ differences could be minimised and their teaching approaches be ‘standardised’. However, there is currently no standardised tool to assess parent’s learning needs and preferences.

MDT members’ described using checklists at certain points with families (e.g. pre-transplant) to ensure that the information given was the same even if the individual HCP’s way of sharing the information differed; however, HCPs acknowledged that the level of information given to the parent could still vary despite the use of checklists. There appeared to be uncertainty amongst HCPs’ about whether to try to ensure teaching and learning encounters were the same with all families, or whether it was more important to adjust the interaction depending on the specific learning needs of the parents, as this interview data suggests:

*“We’re probably bad actually, we don’t have a standardised same thing for everybody, some parents, we assume, need more help and more input; and others, we know they’re very confident and seem to be getting on great with the meds [medicines] and don’t ask any questions or they seem to be just getting on with it. So we probably give the kids that need a bit more help with their meds a bit more time, which is probably the way it should…I don’t know what’s right or what’s wrong to do?”* (Pharmacist_1)

Ambivalence around expertise also appeared to be evident as parents learned how to deliver care. Parents seemed to move along a spectrum of skills, knowledge and confidence; they would move on from gaining competence in delivering essential aspects of a task to making some clinical decisions independently from HCPs. A dietician described during an interview how some families may not follow HCPs’ advice and would adjust care-giving once they got home (for example, a child’s feeds) but acknowledged that parents could be experts because of the experience they had of looking after their child*:*

*“They [parents] do fiddle with the timings [of feeds] and various things and they actually know their child better and it’s just what suits their…you know, it works that way…The sort of group that do never listen to what you say, but, sometimes actually, the changes they make are quite relevant”* (Dietician_4)

Some parents described the experience of developing greater independence, for example one mother who had been learning home dialysis reported during an interview:

*Interviewer: “And making the decisions about increasing her (the child’s) weight, is that something you’re doing on your own?”*

*Mother: “I do it. I just let them (the home dialysis nurse) know what I’ve done next time I speak to them. The nurse will ring and say, ‘what are we up to now?’ (child’s weight). She leaves it very much down to you”*

*Interviewer: “How do you feel about having that responsibility?”*

*Mother: “I was so nervous at the start but I’m fine about it now. Because I can see her [child] and I know the signs when she’s got fluid there. I don’t worry about it because I think, she’s looking a bit puffy, I’ll just stick her on the machine and let’s get rid of it. I don’t worry as much as I used to about it”.*

There appeared to be ambivalence amongst parents about these situations where they seemed to be adopting the role of expert and educator. Some parents accepted the role as they acknowledged HCPs outside of the kidney unit could not be experts in renal care, whereas other parents wanted all HCPs, even those outside of the renal unit, to be able to identify what was wrong with their child and provide treatment, “*instead of me having to explain”.*

The issue of parents knowing when to ask HCPs for advice was discussed by both MDT members’ and parents. Some parents were aware of the limits of their expertise and had developed their own threshold for when they would make a decision independently and when they would seek advice, whereas for some families this threshold was set by the HCP. As keeping the child safe was a priority, MDT members appeared to value parents who exercised caution or were not overconfident in terms of managing their child’s care at home, as reported in this interview:

*“It’s very nice when you can see them become empowered and able to make those steps in their head without you directing them. And obviously, you want the fine balance that you don’t want them running off and doing it all without ever turning to you, but that was never going to be an issue with this family; they would always be cautious”* (Nurse_1)

However, as illustrated by the previous quotation, it was important that a balance be achieved where parents did not consider themselves such experts that they stopped consulting with HCPs. The difficulties of parents having a role where “*they’re part doctor, part nurse, part parent”* was described by HCPs, resulting in some MDT members’ viewing their own role within teaching and learning encounters as supporting parents to be parents ‘first of all’.

## Discussion

Through utilising a focused ethnographic approach which meant spending a prolonged time in the two settings, building trusting relationships with participants and gaining access to situations not often accessible to researchers, we were able to gather data which provide new insights into the interactions between parents of children with CKD stages 3–5 and HCPs in paediatric settings. A systematic review in 2008 [4] recommended that parents of children with CKD (specifically those receiving pre-dialysis care, haemodialysis, peritoneal dialysis, or after kidney transplantation) need multidisciplinary care, which may lead to improved outcomes for their children. Our study addresses this issue using an approach that provided a rich source of data, captured behaviour in everyday contexts, presented real-life stories that HCPs can relate to, and helped to identify discrepancies between what people say they do and what they actually do [25, 43]. Through utilising focused ethnography as we have described in this paper, it was possible to gain a richer understanding of the mutual acknowledgement of expertise, both in terms of multidisciplinary teams of HCPs and parent expertise being shared and blended, but also the ambivalence around expertise experienced by participants.

Two distinct dimensions of parental expertise around their child (personal and clinical) were evident in the data. Personal expertise was often acknowledged at the outset by HCPs who attempted to integrate this into developing the care plan; furthermore, it was evident that HCPs then worked hard to promote parents’ clinical expertise. Parents appreciated HCPs’ recognition of their personal and clinical expertise and sometimes used strategies such as ‘modelling’ to try to replicate the clinical expertise that HCPs demonstrated. Our evidence contradicts the long standing and growing body of literature claiming that there is a lack of collaboration between parents and HCPs, that parents’ expertise is not valued by HCPs and that parent-HCP relationships are characterised by tension and conflict [19, 40, 41, 52]. In a previous qualitative study involving mothers in a children’s kidney unit, the formation of satisfactory alliances with HCPs based on mutual respect and good communication early in the trajectory was instrumental in mothers’ coping and competence development during the later chronic phase of the trajectory [53]. Further research was recommended to map prospectively the evolution of relationships between mothers and staff across the trajectory. The current study has begun to address this recommendation.

Though there was evidence in our data of blended expertise around the child, at times there was some ambivalence around what the expertise was and who was the expert. This supports the findings of Smith et al’s recent review of the evidence which indicates that concepts relating to expertise, including the expert parent, are poorly described [41]. The tensions in defining expertise were also explored by Wilson [35] and could help to explain why we found ambivalence in our study as both parents and HCPs have differing understandings of the concept. Though there is an expectation that an expert has the skills to self-manage their condition, Wilson suggests there is an ‘implicit assumption that the expert is identified as one who knows how to use HCP services appropriately’ [35:136]. This was evident in our study, where HCPs wanted parents to be aware of the limits of their expertise and know when to seek help from the MDT.

The findings reported in this study may be an anomaly of the situation surrounding children with CKD, for instance it may relate to the way in which the responsive network of MDTs in Britain has evolved; or it may be that the design of our study enabled a unique and detailed analysis of the parent teaching and learning context in two children’s kidney units. It is also possible that the presence of the researcher altered the dynamics of interactions in some way, or that our participants’ responses are an anomaly of the sample. The small sample in Phase 3 may not represent all the phenomena that affect parents who use health services. Additionally, the findings may differ from other reported findings because previous studies relied solely on respondents’ retrospective accounts whilst our approach was prospective. Moreover, the research questions posed in previous studies may have focused in particular on participants’ difficulties and negative experiences whereas our approach meant that actual, live interactions between parent and HCPs were observed. Nevertheless, both our study and previous ethnographic studies vividly demonstrate the insights that ethnographic techniques can engender, and confirm the conclusion that they may be better suited than other methods to examining complex interventions in acute and long term child healthcare contexts such as the one we have described here [54, 55].

## Conclusions

Focused ethnography is historically under-utilised in paediatric health settings, yet our findings point to it being an effective way of capturing focused insights into the interactions between parents and HCPs and attitudes towards expertise. It may help redress limitations of previous, retrospective studies, including our own, and therefore, we conclude that studies using focused ethnography have the potential to elicit insights not readily available through other approaches.

## Authors’ information

The authors include researchers and clinicians. RN is an occupational therapist and qualitative researcher with experience in child health; VS is an experienced paediatric nurse with expertise in mixed-methods research and in using Framework Analysis, and MDS is a Paediatric Nephrologist and researcher in a children’s renal unit.
